# Two-year outcomes of treat-and-extend regimen with intravitreal brolucizumab for treatment-naïve neovascular age-related macular degeneration with type 1 macular neovascularization

**DOI:** 10.1038/s41598-023-30146-5

**Published:** 2023-02-24

**Authors:** Hidetaka Matsumoto, Junki Hoshino, Kosuke Nakamura, Hideo Akiyama

**Affiliations:** Department of Ophthalmology, Gunma University Graduate School of Medicine, 3-39-15 Showa-Machi, Maebashi, Gunma 371-8511 Japan

**Keywords:** Macular degeneration, Eye diseases

## Abstract

We previously reported one-year results of a treat-and-extend (TAE) regimen with intravitreal brolucizumab for 68 eyes with treatment-naïve neovascular age-related macular degeneration (nAMD) associated with type 1 macular neovascularization (MNV). In the current study, we evaluated second-year results of the brolucizumab TAE therapy in 45 eyes with type 1 MNV that had completed the first-year treatment. Forty-three eyes (95.6%) received brolucizumab TAE treatment during a period of 96 weeks. The significant improvement of best-corrected visual acuity in the first year was maintained in the second year. Moreover, the significant foveal thickness and central choroidal thickness reductions in the first year were maintained in the second year. The total number of injections over the 96-week study period was 10.0 ± 1.4, with 6.4 ± 0.6 in the first year and 3.6 ± 1.0 in the second year. The intended injection interval at week 96 was 8 weeks in 9 eyes (20.9%), 12 weeks in 3 eyes (7.0%), and 16 weeks in 31 eyes (72.1%), with an average injection interval of 14.0 ± 3.3 weeks. No eyes developed brolucizumab-related intraocular inflammation (IOI) during the second-year treatment. These results indicate that the TAE regimen with intravitreal brolucizumab for treatment-naïve nAMD associated with type 1 MNV effectively maintained the improved visual acuity and the diminished exudative changes in the second year. Moreover, intravitreal brolucizumab has the potential to reduce the treatment burden of nAMD. The risk of developing brolucizumab-related IOI appeared to be very low during the second year of this TAE regimen.

## Introduction

At present, intravitreal injection of an anti-vascular endothelial growth factor (VEGF) agent is the first line treatment for neovascular age-related macular degeneration (nAMD), and this strategy reportedly improves and maintains visual acuity in affected patients^1^. The treatment regimen usually consists of 3 monthly injections in the loading phase, followed by fixed, as needed so-called pro re nata (PRN), or treat-and-extend (TAE) in the maintenance phase^1^. Comprehensive reviews have demonstrated the efficacy of the TAE regimen to be superior to that of the PRN regimen^2^. Moreover, the TAE regimen was shown to be as effective as the fixed regimen, despite the smaller number of injections given^3^. Therefore, TAE is currently the most widely recommended regimen for treating nAMD in practice^1,4^.

The ALTAIR, conducted in Japan, was designed as a 96-week, randomized, open-label, phase 4 study investigating the efficacy and safety of intravitreal injection of aflibercept in patients with treatment-naïve nAMD^5^. The maintenance phase in the ALTAIR study was comprised of two different TAE approaches, i.e., 2-week or 4-week adjustments, with a minimum 8-week and a maximum 16-week interval. These strategies both achieved improvements in functional and anatomic outcomes at week 52 and these outcomes were maintained through week 96. Moreover, the outcomes were similar in the 2- and 4-week adjustment groups. Accordingly, in our hospital, we have adopted a TAE regimen with a minimum 8-week interval, a maximum 16-week interval, and 4-week adjustment as the maintenance phase of anti-VEGF therapy for nAMD patients.

Brolucizumab is a relatively new anti-VEGF agent that was launched commercially following the HAWK and HARRIER studies, both of which were international phase III clinical trials^6,7^. Brolucizumab, with a molecular mass of 26 kDa, is a humanized single-chain antibody fragment characterized by a smaller molecular mass and higher solubility than other anti-VEGF agents^8^. In the HAWK and HARRIER studies, brolucizumab achieved similar visual acuity improvements as well as significantly better controls of intraretinal fluid and/or subretinal fluid, along with subretinal pigment epithelium (RPE) fluid, than aflibercept. However, the incidence of intraocular inflammation (IOI) after injection was found to be higher with brolucizumab than with aflibercept. Although brolucizumab-related IOI can be ameliorated by administering steroid therapy, severe vision loss due to retinal artery occlusion associated with IOI has been reported^9,10^. Therefore, special attention must be paid when using brolucizumab.

We previously reported one-year results of brolucizumab TAE for 68 eyes with treatment-naïve nAMD associated with type 1 macular neovascularization (MNV)^11^. Visual acuity and exudative changes were significantly improved with relatively few visits and injections in 45 eyes (66.2%) that completed the entire one-year treatment regimen. While 15 eyes (22.1%) discontinued brolucizumab treatment due to IOI development, these eyes were treated with aflibercept utilizing PRN or TAE regimens, and visual acuity after 1 year was significantly better than at baseline. Herein, we evaluated the second-year results of the 45 eyes with type 1 MNV that had already completed the first-year brolucizumab TAE therapy.

## Results

From the 45 eyes of 42 patients who completed the first-year treatment with brolucizumab, 2 eyes of 2 patients (2 men, 76 and 83 years) dropped out due to cardiac and brain infarctions. Therefore, subjects analyzed for the second-year results of brolucizumab TAE therapy included 43 eyes of 40 patients (36 eyes of 33 men; 7 eyes of 7 women, age: 72.8 ± 8.2 years) with treatment-naïve nAMD associated with type 1 MNV. Notably, no eyes developed brolucizumab-related IOI during the second-year treatment period.

**Figure 1:**
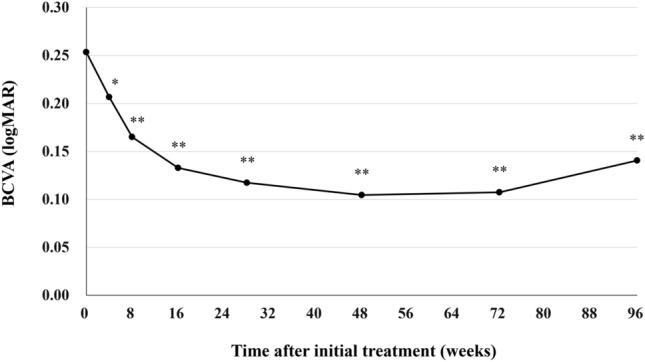
Change of average best-corrected visual acuity (BCVA) in 43 eyes with neovascular age-related macular degeneration associated with type 1 macular neovascularization treated with 3 monthly intravitreal injections of brolucizumab followed by a treat-and-extend regimen with intravitreal brolucizumab. The significant improvement of BCVA in the first year was maintained in the second year. (*P < 0.05, **P < 0.01). Data are expressed as averages. Values at weeks 28, 48, 72, and 96 represent the averages of the values at weeks 24 and 28, 48 ± 4, 72 ± 4, and 84–96, respectively.

**Figure 2:**
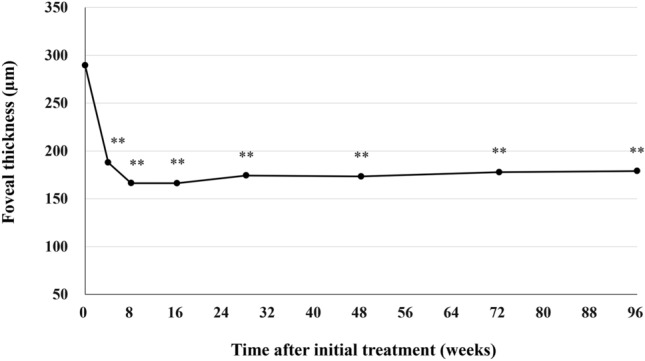
Change of foveal thickness in 43 eyes with neovascular age-related macular degeneration associated with type 1 macular neovascularization treated with 3 monthly intravitreal injections of brolucizumab followed by a treat-and-extend regimen with intravitreal brolucizumab. The significant reduction of foveal thickness in the first year was sustained in the second year. (**P < 0.01). Data are expressed as averages. Values at weeks 28, 48, 72, and 96 represent the averages of the values at weeks 24 and 28, 48 ± 4, 72 ± 4, and 84–96, respectively.

**Figure 3:**
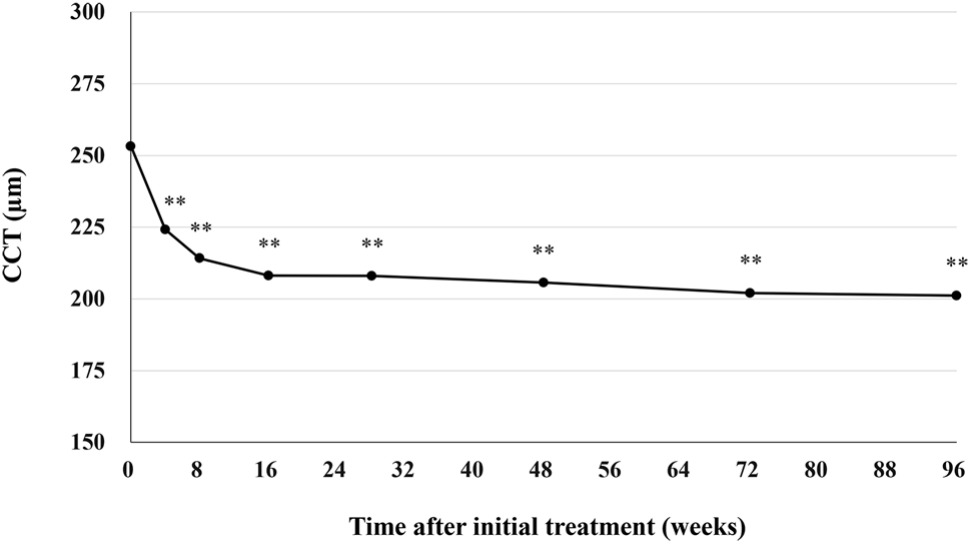
Change of average central choroidal thickness (CCT) in 43 eyes with neovascular age-related macular degeneration associated with type 1 macular neovascularization treated with 3 monthly intravitreal injections of brolucizumab followed by a treat-and-extend regimen with intravitreal brolucizumab. The significant reduction of CCT in the first year was also maintained in the second year. (**P < 0.01). Data are expressed as averages. Values at weeks 28, 48, 72, and 96 represent the averages of the values at weeks 24 and 28, 48 ± 4, 72 ± 4, and 84–96, respectively.

Best corrected visual acuity (BCVA) was 0.25 ± 0.30 at baseline, 0.10 ± 0.26 (*P* < 0.01) at week 48 ± 4, 0.11 ± 0.23 (*P* < 0.01) at week 72 ± 4, and 0.14 ± 0.30 (*P* < 0.01) at week 84–96 (Fig. 1). We excluded one eye from the BVCA analysis in the second year because cataract surgery was performed at the beginning of the second year. The significant improvement of BCVA in the first year was maintained in the second year. BCVA at the last visit was slightly poorer than it had been at week 72 ± 4, since BCVA decreased from 0.40 to 1.52 in one eye showing submacular hemorrhage at the last visit. Foveal thickness was 290 ± 105 at baseline, 173 ± 56 (*P* < 0.01) at week 48 ± 4, 178 ± 55 (*P* < 0.01) at week 72 ± 4, and 179 ± 65 µm (*P* < 0.01) at week 84–96 (Fig. 2). The significant reduction of foveal thickness in the first year was sustained in the second year. Central choroidal thickness (CCT) was 253 ± 91 at baseline, 206 ± 80 (*P* < 0.01) at week 48 ± 4, 202 ± 78 (*P* < 0.01) at week 72 ± 4, and 201 ± 81 µm (*P* < 0.01) at week 84–96 (Fig. 3). The significant CCT reduction documented during the first year was also maintained in the second year.

**Figure 4:**
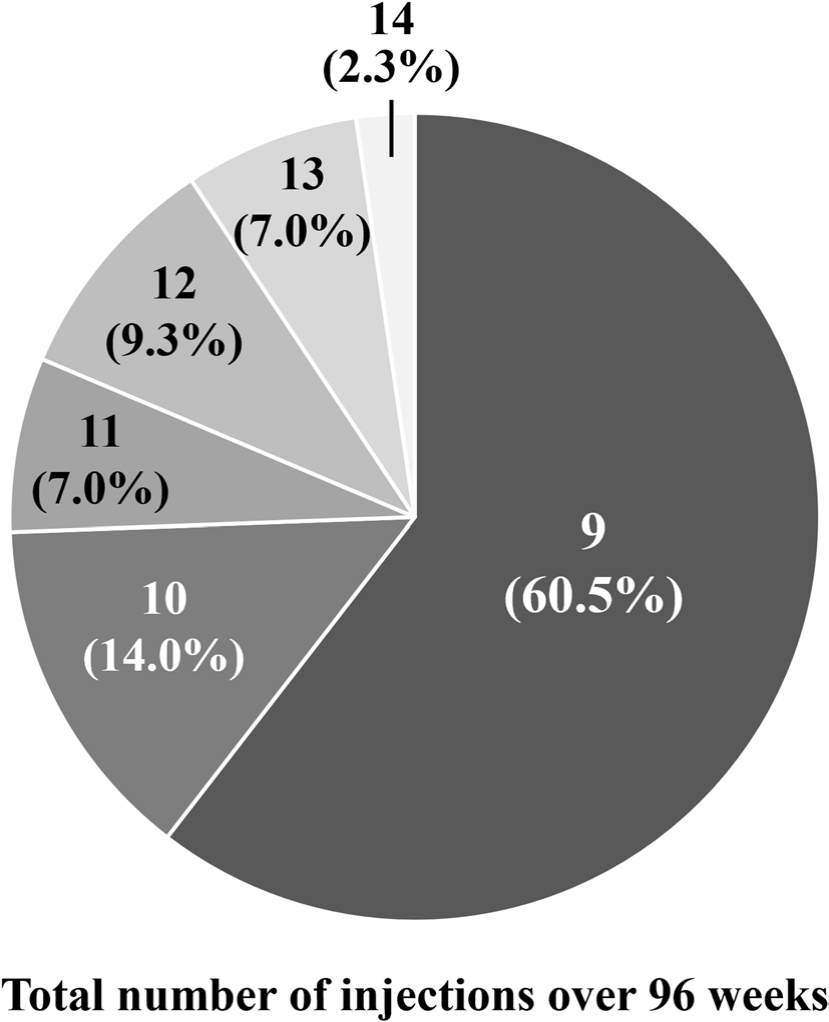
Total number of injections over the 96-week study period in 43 eyes with neovascular age-related macular degeneration associated with type 1 macular neovascularization treated with 3 monthly intravitreal injections of brolucizumab followed by a treat-and-extend regimen with intravitreal brolucizumab.

**Figure 5:**
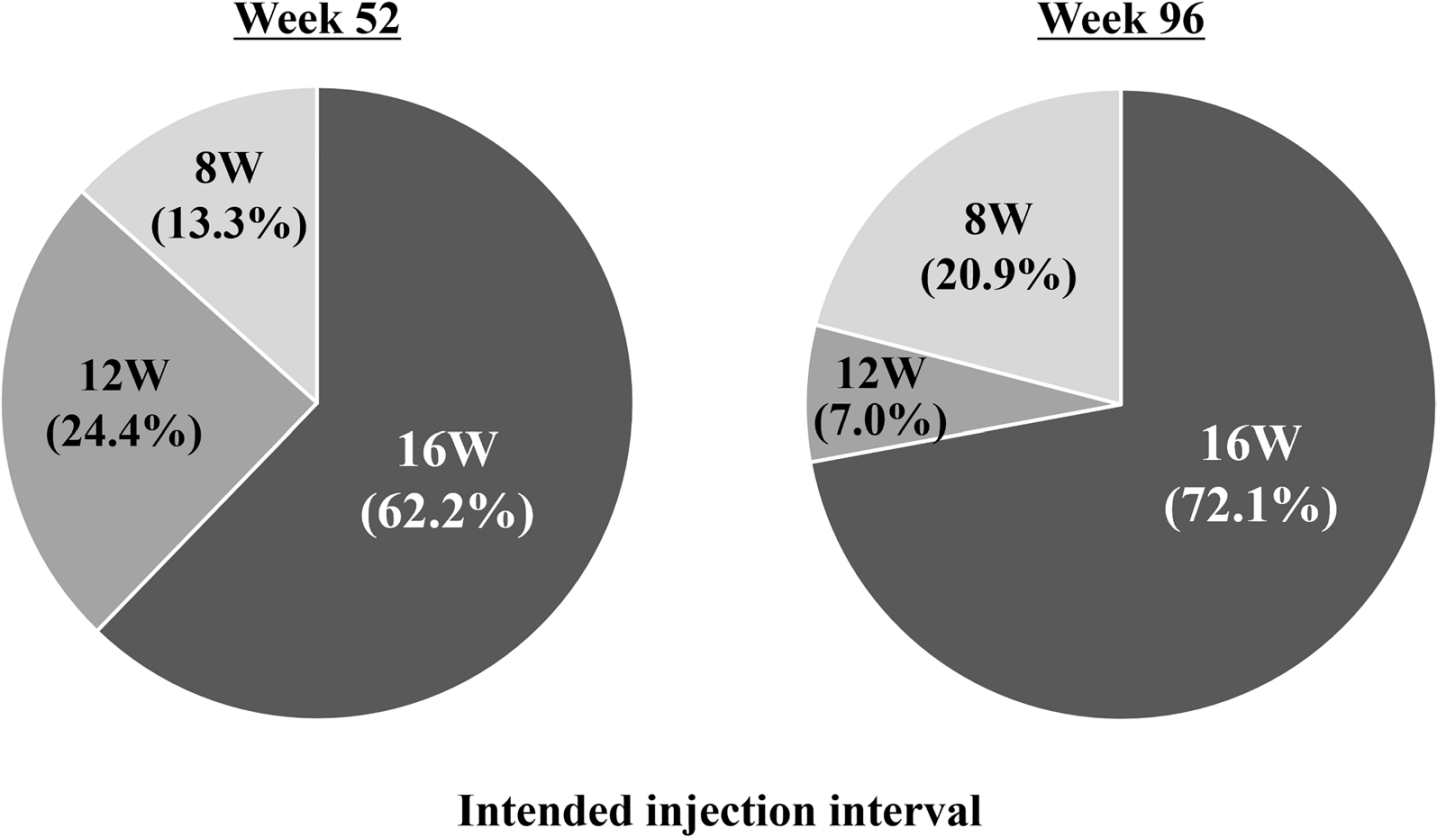
Intended injection intervals at weeks 52 and 96 in 43 eyes with neovascular age-related macular degeneration associated with type 1 macular neovascularization treated with 3 monthly intravitreal injections of brolucizumab followed by a treat-and-extend regimen with intravitreal brolucizumab.

The total number of injections over the 96-week study period was 10.0 ± 1.4, with 6.4 ± 0.6 in the first year and 3.6 ± 1.0 in the second year (Fig. 4). Twenty eyes (46.5%) required the minimum number of 9 injections and experienced no recurrence of exudative changes through week 96. The intended injection interval at week 52 was 8 weeks in 6 eyes (13.3%), 12 weeks in 11 eyes (24.4%), and 16 weeks in 28 eyes (62.2%), with an average injection interval of 14.0 ± 2.9 weeks. At week 96, it was 8 weeks in 9 eyes (20.9%), 12 weeks in 3 eyes (7.0%), and 16 weeks in 31 eyes (72.1%), with an average injection interval of 14.0 ± 3.3 weeks (Fig. 5). Comparison of the results at 52 and 96 weeks revealed that the rate of the 12-week interval decreased while that of the 8- and 16-week intervals increased (*P* < 0.01).

Twenty-two eyes (51.2%) in this study showed type 1 MNV with polypoidal lesions. We compared the 96-week outcomes between the eyes with and without polypoidal lesions. However, there were no significant differences in BCVA change (− 0.06 ± 0.18 vs − 0.09 ± 0.29, P = 0.39), the total number of injections (14.5 ± 2.8 vs 13.5 ± 3.6, P = 0.37), or the intended injection interval at week 96 (9.7 ± 1.3 vs 10.2 ± 1.5, P = 0.28).

The total number of injections for the 96-week study period was significantly lower in eyes that showed dry macula at week 16 than in the other cases (9.7 ± 1.1 vs. 11.4 ± 1.9, *P* < 0.05). Moreover, the intended injection interval at week 96 was significantly longer in those with dry macula at week 16 than in the other eyes (14.6 ± 2.9 vs 11.4 ± 4.0 weeks, *P* < 0.05) (Table 1). The total number of injections during the 96-week study period was significantly lower in cases showing complete regression of polypoidal lesions after the loading phase than in the other patients (9.3 ± 0.8 vs 10.8 ± 1.7, *P* < 0.05). However, the intended injection intervals at week 96 did not differ significantly between the two groups (14.8 ± 2.7 vs 14.0 ± 3.1, P = 0.51) (Table 2).

**Table 1:** Comparison between eyes with and without dry macula at week 16.

	Dry macula ( +)	Dry macula (–)	*P* value
Number of eyes	36 (83.7%)	7 (16.3%)	
Number of injections during the 96-week study period	9.7 ± 1.1	11.4 ± 1.9	0.02
Intended injection interval at week 96 (weeks)	14.6 ± 2.9	11.4 ± 4.0	0.04

**Table 2:** Comparison between eyes with and without complete regression of polypoidal lesions after the loading phase treatment.

	Complete regression of polypoidal lesions ( +)	Complete regression of polypoidal lesions (–)	*P* value
Number of eyes	16 (72.7%)	6 (27.3%)	
Number of injections during the 96-week study period	9.3 ± 0.8	10.8 ± 1.7	0.02
Intended injection interval at week 96 (weeks)	14.8 ± 2.7	14.0 ± 3.1	0.51

In the analysis of macular atrophy (MA), one eye was excluded because of extensive subretinal hemorrhage at baseline and at the last visit. MA was present in 6 of 42 eyes (14.3%) at baseline, and the MA area expanded in 4 eyes (66.7%) during the 96-week study period. Among 36 eyes without MA at baseline, 3 eyes (8.3%) developed MA during the 96-week treatment course.

We compared baseline characteristics, including age, gender, BCVA, foveal thickness, CCT, presence of polypoidal lesions, presence of MA, and greatest linear dimension of MNV, between the eyes with an 8- or 12-week intended injection interval and those with a 16-week intended injection interval at week 96. Foveal thickness was found to be significantly higher in eyes with the 8- or 12-week injection interval than in eyes with the 16-week injection interval (355 ± 110 µm vs 267 ± 94 µm, *P* < 0.05), while other baseline parameters did not differ significantly between the two groups (Table 3).

**Table 3:** Comparison of baseline characteristics between eyes with an 8- or 12-week intended injection interval versus those with a 16-week intended injection interval at week 96.

	Total	Intended injection interval at week 96		*P* value
		8 or 12 weeks	16 weeks	
Number of eyes	43	12	31	
Number of patients	40	10	30	
Age (years)	72.8 ± 8.2	72.3 ± 8.2	72.9 ± 8.2	0.99
Male	33 (82.5%)	8 (80.0%)	25 (83.3%)	0.81
Best-corrected visual acuity (logMAR)	0.25 ± 0.30	0.28 ± 0.29	0.22 ± 0.28	0.56
Foveal thickness (µm)	290 ± 105	355 ± 110	267 ± 94	0.02
Central choroidal thickness (µm)	253 ± 91	251 ± 96	254 ± 88	0.92
Greatest linear dimension of macular neovascularization (µm)	2311 ± 1122	2952 ± 1425	2070 ± 906	0.09
Polypoidal lesions ( +)	22 (51.2%)	5 (41.7%)	17 (54.8%)	0.44
Macular atrophy ( +)	6 (14.3%)	2 (18.2%)	4 (12.9%)	0.75

## Discussion

We investigated second-year results of a TAE regimen with intravitreal brolucizumab for 43 eyes with treatment-naïve nAMD associated with type 1 MNV that had completed the entire first year of treatment^11^. The significant improvement of BCVA as well as the significant decreases in foveal thickness and CCT in the first year were maintained during the second year. Twenty eyes (46.5%) required the minimum number of 9 injections and experienced no recurrence of exudative changes through week 96. At week 96, 31 eyes (72.1%) had been given the intended maximum injection interval of 16 weeks. No eyes developed brolucizumab-related IOI during the second-year treatment period.

In the present study, the total number of injections over the 96-week study period was 10.0 ± 1.4 and the intended injection interval at week 96 was 14.0 ± 3.3. On the other hand, in the 4-week adjustment group of the ALTAIR study, the total number of injections was 10.4 ± 2.4 and the intended injection interval was 12.5 ± 3.6 weeks^5^. The ALTAIR study targeted nAMD with all subtypes of MNV^5^, whereas our study targeted nAMD associated with type 1 MNV, in which it is relatively challenging to control exudation with intravitreal injection of anti-VEGF agents^12^. Moreover, the injection interval was maintained if the fluid was decreasing in the ALTAIR study^5^, while it was shortened by 4 weeks if even a small amount of fluid was detected in the present study. Taken together, these results suggest that brolucizumab might be superior to aflibercept in terms of fluid control.

In the current study, the cases that achieved dry macula at week 16 had significantly fewer injections during the 96-week study period and a significantly longer intended injection interval at week 96 than the other cases. In the ALTAIR study, a greater percentage of patients without, as compared to those with, fluid at week 16 achieved the maximum treatment interval of 16 weeks^13^. Furthermore, consistent with our previous reports^11,14^, the total number of injections for the 96-week study period was significantly lower in cases showing complete regression of polypoidal lesions after the loading phase than in the other patients. Thus, the response to the loading phase treatment may allow us to estimate two-year outcomes.

The proportion of patients with the intended brolucizumab injection interval of 16 weeks rose from 62.2% at week 52 to 72.1% at week 96 in the present study. It was also increased from 40.7% at week 52 to 46.3% at week 96 in the 4-week adjustment group of the ALTAIR study^5^. Moreover, similar results were obtained in the TENAYA and LUCERNE clinical trials evaluating the efficacy and safety of faricimab for nAMD^15^. In these studies, faricimab was administered in 4 monthly injections, followed by a fixed dose based on disease activity until week 60, and then by applying a personalized treatment interval until week 112. The respective minimum and maximum injection intervals were 8 and 16 weeks. In the TENAYA and LUCERNE studies, the proportions of patients with the intended injection interval of 16 weeks rose from 45.7% and 44.9% at week 48 to 59.0% and 66.9% at week 112, respectively^15^. These results suggest that in some cases, long-term use of anti-VEGF agents can reduce nAMD disease activity and prolong the injection interval. On the other hand, in all of these studies, there was a certain percentage of patients for whom fluid control was insufficient even with an 8-week injection interval^5,15^. Switching to other anti-VEGF agents or photodynamic therapy should probably be considered in such cases because of the risk of long-term vision loss^16^.

We sought to identify baseline characteristics possibly affecting the response to brolucizumab TAE therapy. In our analysis, baseline foveal thickness was found to be significantly higher in eyes with an 8- or 12-week intended injection interval than in eyes with a 16-week intended injection interval at week 96. This result suggests that higher MNV activity and/or RPE damage might result in greater foveal thickness at baseline and a poorer response to brolucizumab therapy, although further studies with more cases are needed.

Marked suppression of ocular VEGF may lead to the development of MA. In this study, 4 of 6 eyes (66.7%) with MA at baseline showed expansion of the area, and 3 of 36 eyes (8.3%) without MA at baseline developed MA during the 96-week study period. Kuroda et al. reported that 9 of 135 eyes (6.7%) with typical AMD or PCV showed MA at baseline, and that 13 of 123 eyes (10.6%) developed MA after 12 months of aflibercept therapy utilizing 3 monthly injections followed by bi-monthly injections^17^. The incidence of MA during anti-VEGF therapy might be affected by several factors besides the type of anti-VEGF agent, including MNV type, disease activity and duration, and the dosing regimen. Therefore, further studies are needed to clarify the risk of MA development specific to each anti-VEGF agent.

In our previous study evaluating the first-year results of brolucizumab TAE for treatment-naïve nAMD with type 1 MNV, brolucizumab-related IOI developed in 15 of 68 eyes (22.1%), 14 (93.3%) of which showed IOI findings within the first 3 months of treatment initiation^11^. However, none of the eyes in the present study, evaluating the second-year results, showed indications of IOI at any timepoint. Post hoc analysis of the HAWK and HARRIER studies also revealed that the incidence of IOI peaked within 3 months of the first brolucizumab injection^18^. Moreover, there were no IOI cases with moderate or severe visual acuity loss in the second year of the HAWK and HARRIER studies^18^. Therefore, the cases completing the first-year treatment with brolucizumab without IOI might be at low risk of developing IOI and IOI-related vision loss.

When treating patients with intravitreal injections of anti-VEGF agents, it is necessary to remain aware of possible systemic effects. A study examining blood VEGF levels after intravitreal injection of aflibercept or brolucizumab revealed a significant decrease in blood VEGF-A levels 7 days after an aflibercept injection, but levels had recovered to the baseline at 28 days post-injection^19^. In contrast, brolucizumab was associated with a significant decrease in blood VEGF-A levels both 7 and 28 days after the injection, suggesting a possible long-term decrease in blood VEGF-A levels with brolucizumab injection^19^. However, the HAWK and HARRIER studies reported no significant difference in the incidence of non-ocular adverse events including cardiac and vascular disorders between the aflibercept and brolucizumab groups^6^. In our study, 2 patients dropped out, one due to a cardiac and the other to a brain infarction, during the second year of treatment with brolucizumab. Although the associations between these adverse events and brolucizumab injections remain uncertain, careful administration is recommended for patients at high risk of vascular events.

The limitations of our study include its retrospective single-center design, the small number of patients and the lack of controls. Eyes with nAMD with type 2 or type 3 MNV were not included in this study. All of our subjects were Japanese, such that the results may not be generalizable to nAMD in Caucasians and other groups. To our knowledge, this is the first report evaluating 2-year real-world data obtained from intravitreal brolucizumab therapy for nAMD. However, longer term outcomes need to be assessed because nAMD is a chronic disease.

In conclusion, the second year of a TAE regimen with intravitreal brolucizumab for treatment-naïve nAMD associated with type 1 MNV effectively maintained the improvements in visual acuity and exudative changes that had been obtained in the first year. Moreover, relatively few visits and injections were required for this treatment. No eyes developed brolucizumab-related IOI during the second year of our TAE regimen.

## Methods

We obtained approval from the Institutional Review Board of Gunma University Hospital and adhered to the guidelines of the Declaration of Helsinki in performing this study. Informed consent was obtained from all individual participants included in the study. We retrospectively studied second-year outcomes in 45 eyes of 42 patients with treatment-naïve nAMD associated with type 1 MNV that had completed the first year of brolucizumab TAE therapy. During the period from June 2020 through January 2021, the patients started to receive 3 monthly intravitreal injections of brolucizumab as a loading phase followed by a TAE regimen with intravitreal brolucizumab as a maintenance phase at Gunma University Hospital^11,20^. All eyes evaluated in this study were included in our previous investigation of one-year outcomes of brolucizumab TAE therapy for type 1 MNV secondary to nAMD^11^.

Before starting the treatment with intravitreal brolucizumab, all patients underwent complete ophthalmological examinations, including slit-lamp biomicroscopy with a noncontact fundus lens (SuperField lens; Volk Optical Inc, Mentor, OH), color fundus photography and fundus autofluorescence (FAF) (Canon CX-1; Canon, Tokyo, Japan), ultra-widefield color fundus imaging (Optos 200Tx, Optos, Dunfermline, UK), fluorescein angiography (FA) and indocyanine green angiography (ICGA) (Spectralis HRA + OCT; Heidelberg Engineering, Heidelberg, Germany), as well as swept-source optical coherence tomography (OCT) (DRI OCT-1 Triton; Topcon Corp, Tokyo, Japan, and PLEX Elite 9000; Carl Zeiss Meditec, Dublin, CA, USA). In the OCT examination, we obtained B-mode images of the horizontal and vertical line scans (12 mm) through the fovea employing the DRI OCT-1 Triton. Then, we performed OCT angiography (OCTA) volume scanning, i.e., 300 × 300 pixels in the 3 × 3 mm area demonstrated by the PLEX Elite 9000. The OCTA thus performed was based on an optical microangiography algorithm. The diagnostic criteria for nAMD were based on a previous report of nAMD nomenclature^21^. We diagnosed nAMD with type 1 MNV, if MNV was detected beneath the RPE regardless of the presence of polypoidal lesions by the aforementioned multimodal imaging. The greatest linear dimension of MNV was determined based on the ICGA and OCTA findings.

All eyes were treated with only intravitreal brolucizumab injection (6 mg/0.05 mL). In the loading phase, patients receive 3 monthly injections of brolucizumab. All patients again underwent FA and ICGA at week 12, i.e., 4 weeks after the third brolucizumab injection. In the maintenance phase, the interval of injections is extended by 4 weeks if there are no exudative changes, whereas the interval is shortened by 4 weeks in the event of any exudative change being detected. Herein, we set the treatment interval at a minimum of 8 weeks and a maximum of 16 weeks. Moreover, the study period was set to end at week 96.

At every visit, we determined BCVA, as well as performing slit-lamp biomicroscopy with a noncontact fundus lens, color fundus photography, FAF, ultra-widefield color fundus imaging, and swept-source OCT examinations. BCVA was determined with manifest refraction and recorded as decimal values and then converted to the logarithm of the minimal angle of resolution (logMAR) units. Foveal thickness and CCT were measured on B-scan OCT images employing the computer-based caliper measurement tool in the OCT system. Foveal thickness was, by definition, the distance between the internal limiting membrane and the RPE surface at the fovea. Foveal thickness included any intraretinal and subretinal fluid. CCT was defined as the distance between Bruch’s membrane and the margin of the choroid and sclera under the fovea. Dry macula was defined as the absence of intraretinal, subretinal, and sub-RPE fluid accompanied by either no or diminishing hemorrhage. As reported previously^17^, the diagnosis of MA required that the following five conditions be met: 1) its presence within the macular vascular arcade; 2) a roughly round or oval area of partial or complete depigmentation of the RPE, with thinning of the overlying neurosensory retina; 3) ≧250 mm in the longest linear dimension; 4) atrophic changes in the RPE and photoreceptor cells with increased choroidal signal beneath them both on OCT; and 5) at least one of the following additional characteristics: sharp demarcated borders, visibility of underlying choroidal vessels, or a uniformly reduced autofluorescence signal bounded by sharp borders on FAF.

For statistical analyses, the Wilcoxon signed-rank test was employed for comparison of the differences between BCVA, foveal thickness and CCT at baseline versus other timepoints. The Chi-square test was applied to determine the difference in intended injection intervals between week 52 and week 96, as well as the differences in gender, the presence of polypoidal lesions, and the presence of MA between the eyes with an 8- or 12-week intended injection interval and those with a 16-week intended injection interval at week 96. Unpaired values for age, BCVA, foveal thickness, CCT, greatest linear dimension of MNV, number of injections, and intended injection interval were compared using the Mann–Whitney U test. The data analyses were performed using Excel (Microsoft, Redmond, WA, USA) with add-in software Statcel4^22^. A *P* < 0.05 was considered to indicate a statistically significant difference. All data are presented as the average ± standard deviation.

## Data Availability

The datasets used and/or analyzed during the current study are available from the corresponding author upon reasonable request.
